# Genetic and Environmental Influences on the Relationship between Flow Proneness, Locus of Control and Behavioral Inhibition

**DOI:** 10.1371/journal.pone.0047958

**Published:** 2012-11-02

**Authors:** Miriam A. Mosing, Nancy L. Pedersen, David Cesarini, Magnus Johannesson, Patrik K. E. Magnusson, Jeanne Nakamura, Guy Madison, Fredrik Ullén

**Affiliations:** 1 Department of Neuroscience, Karolinska Institutet, Stockholm, Sweden; 2 Department of Medical Epidemiology and Biostatistics, Karolinska Institutet, Stockholm, Sweden; 3 Center for Experimental Social Science, Center for Neuroeconomics and Department of Economics, New York University, New York, New York, United States of America; 4 Research Institute of Industrial Economics, Stockholm, Sweden; 5 Department of Economics, Stockholm School of Economics, Stockholm, Sweden; 6 Quality of Life Research Center, Claremont Graduate University, Claremont, California, United States of America; 7 Department of Psychology, Umeå University, Umeå, Sweden; George Mason University/Krasnow Institute for Advanced Study, United States of America

## Abstract

*Flow* is a psychological state of high but subjectively effortless attention that typically occurs during active performance of challenging tasks and is accompanied by a sense of automaticity, high control, low self-awareness, and enjoyment. Flow proneness is associated with traits and behaviors related to low neuroticism such as emotional stability, conscientiousness, active coping, self-esteem and life satisfaction. Little is known about the genetic architecture of flow proneness, behavioral inhibition and locus of control – traits also associated with neuroticism – and their interrelation. Here, we hypothesized that individuals low in behavioral inhibition and with an internal locus of control would be more likely to experience flow and explored the genetic and environmental architecture of the relationship between the three variables. Behavioral inhibition and locus of control was measured in a large population sample of 3,375 full twin pairs and 4,527 single twins, about 26% of whom also scored the flow proneness questionnaire. Findings revealed significant but relatively low correlations between the three traits and moderate heritability estimates of .41, .45, and .30 for flow proneness, behavioral inhibition, and locus of control, respectively, with some indication of non-additive genetic influences. For behavioral inhibition we found significant sex differences in heritability, with females showing a higher estimate including significant non-additive genetic influences, while in males the entire heritability was due to additive genetic variance. We also found a mainly genetically mediated relationship between the three traits, suggesting that individuals who are genetically predisposed to experience flow, show less behavioral inhibition (less anxious) and feel that they are in control of their own destiny (internal locus of control). We discuss that some of the genes underlying this relationship may include those influencing the function of dopaminergic neural systems.

## Introduction

*Flow* is a psychological state of high but subjectively effortless attention accompanied by a sense of automaticity, high control, low self-awareness, and enjoyment. It typically is experienced during the performance of challenging activities that are matched in difficulty to one's skill level [1], [2], i.e. if a task is very easy (below the skill level of the individual) it will be perceived as boring and if it is very difficult (beyond the skill level of an individual) it will be perceived as stressful, rather than inducing flow. Several studies have shown that the flow experience is associated with high levels of performance, objectively measured [1], [3]. Flow can occur in a wide range of activities and settings, but there are large individual differences in how prone people are to experience flow in daily life [2], [4], [5]. A high self-reported flow proneness is associated with other positive outcomes, such as psychological well-being, self-esteem, life satisfaction and active coping strategies [3], [5]–[7]. Flow proneness has also been shown to be related to personality. Ullén et al. [8] recently reported a positive relationship between flow proneness and conscientiousness and a negative association with neuroticism. The latter finding is further supported by earlier studies reporting negative associations between flow proneness and traits related to neuroticism such as trait anxiety [5], [9].

Behavioral inhibition, a stable temperamental trait characterized by fearful reactivity to novelty, has also repeatedly been associated with internalizing disorders such as anxiety [10]–[14] and neurotic introversion [15], [16], as well as social withdrawal and avoidant behavior [17]. Given the importance of the challenge/skill balance for a flow experience – the difficulty of the task has to be matched to the skill level of a person – we hypothesized that behavioral inhibited individuals may be less likely to actively seek out situations where their skill-level may be challenged and therefore may be less likely to enter a flow state. As mentioned above, this is further supported by the finding that flow-proneness is negatively related to trait-anxiety [5], [9], which in turn is positively related to behavioral inhibition [14].

It has also been argued that the enjoyment associated with a flow experience may serve as a reward signal important for intrinsic motivation to perform a task [1], [18], [19]. This suggests that personality traits related to an internal locus of control [20] – a belief that rewards are dependent upon one's own behavior rather than external forces – increase the probability of experiencing flow. Indeed, it has been shown that an internal locus of control is associated with an increased sensitivity to skill-demand compatibility when inducing flow under experimental conditions but also with a higher likelihood to enter a flow state when skill-demand compatibility is met [21]. Also, more generally, an internal locus of control has been associated with personality correlates of flow proneness, i.e. high conscientiousness and low neuroticism [22]. It is also, like flow proneness, related to active task engagement [20], active coping strategies [23], life satisfaction [24], psychological well-being [25] and positive mood [24].

Finally, personality as well as flow proneness has been shown to be moderately heritable. While the heritability of personality – typically reported to range between .4 and .6 - has been established by numerous twin studies [26]–[29], we were the first to explore genetic influences on the proneness to experience flow [30]. Our data revealed heritability estimates of .29, .35, and .33 for flow proneness during leisure, maintenance (i.e. personal and household care) and work activities, respectively. One shared genetic factor explained the genetic variance of flow proneness in all three domains as well as the vast majority of the covariance between the three domains [30], suggesting that the same set of genes influences flow proneness independently of domain, while within-individual differences in proneness to flow in different domains are likely due to specific environmental influences. Although locus of control appears to be one of the most widely studied traits [22] and also behavioral inhibition perceived a lot of attention, only very few studies have explored genetic and environmental influences underlying those two constructs. To our knowledge only two studies – one very small family study [31] and one twin/adoption study utilizing older twins [32] – have explored the genetic architecture of locus of control and only one twin study has explored genetic influences on the behavioral inhibition system (BIS) in adults [33], [34]. All studies reported heritability estimates of about .30.

Here, we aimed to explore the genetic architecture of the relationship between flow proneness (FP), behavioral inhibition (BI) and locus of control (LOC), utilizing a genetically informative sample of adult twins.

## Methods

### Participants

The present study used data from a recent and extensive web-based survey – the SALTY study – sent out to approximately 25,000 twins born between 1943 and 1958 registered with the Swedish Twin Registry (STR), one of the largest registries of its kind. Participation rate was about 45%–11,369 individuals – of those 92 individuals had a missing zygosity score and were therefore deleted, leaving 11,277 individuals – 3,375 full pairs and 4,527 single twins aged between 51 and 66 (mean 58.9, SD 4.6). For further details on the present sample and a comparison of respondents to non-respondents on a number of background characteristics see Dawes et al. [35]. In the questionnaire, participants were asked to fill in an additional online survey (login-name and password was supplied), which was done by a subsample of 2,937 (444 full pairs and 2,049 single twins). While BI and LOC was part of the original paper-based questionnaire, flow-proneness was only included in the online questionnaire, resulting in a much smaller N for this trait (for demographic information of the paper-based and the online sub-sample see Table S1). Single twin-individuals were retained for analysis as they contribute to the estimation of means, variances, and covariate effects. Zygosity was assigned based on questions about intra-pair similarities in childhood; in 27% of the twins in the STR, zygosity has been determined using genotyping [36]. In the STR, zygosity validation based on genotyping has repeatedly shown a high accuracy (more than 98% correct) of the traditional zygosity determination based on twin-similarity [36].

### Ethics Statement

The study was approved by the Regional Ethical Review Board in Stockholm (Dnr 2008/1735-31/3). All participants have given written and informed consent.

### Measures

*The Swedish Flow Proneness Questionnaire (SFPQ)* was used as an indicator for flow proneness in daily life. The SFPQ is a 22-item self-report measure developed to estimate an individual's proneness to experience flow [8] and consists of three sub-scales (seven items each) assessing FP during work, leisure, and maintenance activities. One additional item assesses current employment as the work sub-scale was administered only to working individuals. A typical question would be: “When you do something at work, how often does it happen that you feel completely concentrated?” Each item had the following five response options: “Never” (1), “Rarely” (2), “Sometimes” (3), “Often” (4), and “Every day, or almost every day” (5). Here, based on previous findings of one common genetic factor explaining the entire genetic variance in the three flow domains [30], the total average scale score provided an estimate of overall FP independent of domain. The SFPQ has been shown to possess a relatively high construct validity and internal consistency (Cronbach's α = .83). For further details on the SFPQ and the present sample see Ullén et al. [8] and Mosing et al. [30].

*The Locus of Control Scale (LOC)* was used to assess locus of control. The LOC has been developed to provide an estimate of an individual's perception of control over event outcomes [20]. Here, the reduced 13 item version of the original LOC scale [20] was used. Each item consists of two statements about the “nature of the world” (one representing externality and one representing internality) and the participant chooses the one most representative of his/her own beliefs. A sample item is: (a) Many of the unhappy things in people's lives are partly due to bad luck; (b) People's misfortunes result from the mistakes they make. The total score then is the number of external choices made. One of the items was not included in the survey given the middle aged sample as it addressed students; therefore, the final LOC score in the present study ranged between 0 and 12. Here, we reverse-coded the scale. Therefore, high score on the LOC indicates an *internal* locus of control which is associated with a feeling of control over one's own destiny and the belief that outcomes in life are a result of one's own skills, behaviors, and efforts. On the contrary, a low score on the LOC indicates an *external* locus of control which is associated with the feeling that outcomes in live are beyond one's control and mainly attributable to external influences.

*The Adult Measure of Behavioral Inhibition (AMBI)* was used to assess behavioral inhibition. The AMBI [37] consists of 16 items assessing an individual's general long-standing inhibition in response to social novelty and risk stimuli. An example question of the AMBI would be: “When you enter a new or unfamiliar social situation or whenever you are faced with new and unfamiliar surroundings or people do you tend to introduce yourself to new people?” Items were rated on a 3-point scale, i.e. “no/hardly ever” (0), “some of the time” (1), “yes/most of the time” (2). Here, the AMBI was reverse-coded so that a low score reflects avoidant and introverted behavior (high inhibition), while a high score is associated with low inhibition (extraversion). Scores in the present sample ranged between 0 and 31.

### Statistical analyses and genetic modeling

The three variables were converted to z-scores with a mean of zero and a standard deviation of one. Scores more than three standard deviation below or above the mean were winsorized – 22 individuals for LOC and 14 individuals for BI and FP, respectively [38]. Analyses were conducted utilizing maximum-likelihood modeling in the statistical program Mx [39], [40]. In Mx all variables are assumed to be continuous and normally distributed. Means and variances were constrained equal across twins and all zygosity groups, while the twin correlations between zygosity groups were allowed to differ. Twin correlations for the two zygosity groups – monozygotic (MZ) versus dizygotic (DZ) – and for all five zygosity groups – MZ female, MZ male, DZ female, DZ male, and DZ opposite-sex – were estimated. Subsequently, genetic models were specified in which individual differences (i.e. the phenotypic variance and covariance) were modeled as a function of genetic (additive genetic (A), dominant genetic (D)) and environmental influences (shared environmental (C), and non-shared environmental (E)). The latter (E) includes measurement error. The classical twin design utilizes the fact that, while DZ twins on average only share half their segregating genes, MZ twins share their entire genome. This knowledge allows for the prediction of the model (A, C, D, and E influences) best fitting the observed MZ and DZ twin correlations. However, as C and D are negatively confounded – C decreases the MZ-DZ correlation ratio, while D tends to increase it – only three of the four sources of variance can be considered (i.e. ADE or ACE models). When DZ twin correlations are at least half the MZ correlation, shared environmental influences are implied and, generally, an ACE model is fitted. If DZ twin correlations are less than half the MZ correlations though, dominant genetic influences are implied and an ADE model is more appropriate. The significance of specific parameters and, therefore, the compatibility of different models to the data was tested by comparing the fit of nested and increasingly more reduced models to the fit of the full or less restricted models. The goodness-of-fit (−2LL) of a model to the data, which follows a χ2 distribution, can be compared against the change in degrees of freedom (Δdf). If the change in goodness-of-fit (Δ −2LL) is not significant, generally, the more parsimonious model is regarded as the one of choice. Here, a criterion level of α = 0.05 was adopted for all tests.

A trivariate ADE Cholesky decompositions was fitted initially, with FP, BI, and LOC. Then, the model fit of the reduced models (AE, DE, and E models) was compared to the full or less restricted models to determine the most parsimonious model. Finally, a trivariate GE model was fitted, to estimate the total genetic effect (broad-sense heritability) with confidence intervals [41], [42]. The correlation between estimates of A and D is very high, resulting in a low reliability compared to estimates of broad-sense heritability [G]; [ 43,44]. Instead of fixing the genetic relationship between DZ twins to either 0.5 (A) or 0.25 (D), in a GE model it is left free to range between 0 and 0.5, taking epistasis into account which can result in a genetic relationship of up to zero [41]. This allows for the estimation of confidence intervals for the total genetic influences (rather than for A and D separately) on the variance and covariance of the traits, which is of particular interest when there is a lack of power to distinguish between A and D influences, resulting in non-significant estimates for both.

## Results

### Preliminary analyses

All three traits were normally distributed. The covariates age and sex had a significant effect on BI with females and older people being less impulsive (more inhibited). Sex also had a significant effect on LOC and FP with women being more likely to have an external LOC (low score) and reporting slightly higher flow proneness. In order to control for participation bias, we checked whether the means for the variables were different between those individuals who only did the paper-based survey and those who also filled in the online survey [45], [46]. The analysis revealed that there was no significant difference in means (LOC and BI) and variances between the two participant groups. However, not unexpectedly, there were small but significant age and sex differences between the two sub-samples, with the online sample being slightly younger and more likely to be male (Table S1).

Means and standard deviations for males and females for all three variables are shown in Table 1. The phenotypic correlations (Table 2) were relatively low but significant ranging between .18 (FP and LOC) and .23 (LOC and BI). Twin correlations (summarized in Table 2) were moderate for MZ twins – ranging between .29 and .45 – while the DZ correlations were much lower – ranging between .09 and .14 – and non-significant for FP. The confidence intervals indicated that the MZ correlations were significantly higher than the DZ correlations for all variables except FP. All MZ correlations were more than twice the DZ correlations indicating the presence of dominant genetic effects. Finally, twin correlations for the five zygosity groups (Table 2) showed no indication for sex differences for FP (all CIs were very wide and overlapping suggesting low power to detect sex differences) and LOC (DZ correlations ranging between .06–.12 with overlapping CIs and both MZ correlations being close to .30). MZ twin correlations for BI were significantly lower for males (.37) than for females (.51), while the CIs for DZ correlations were all overlapping, indicating potential differences in heritability between the sexes. However, given the small N for FP, we had not enough power to model the five zygosity groups separately in the multivariate analysis. Therefore, an additional univariate sex-limitation model for BI was conducted to explore potential sex differences in heritability. Given that the DZ opposite-sex correlations were not significantly lower than the DZ same-sex correlations we only included same-sex pairs in the model.

**Table 1:** Means and standard deviations for the three variables for females and males separately and for the total sample.

Mean (SD)	Flow Proneness	Behavioral Inhibition	Locus of Control
Females	3.83 (0.44)	17.89 (5.09)	6.54 (2.09)
Males	3.78 (0.41)	18.03 (4.93)	6.83 (2.15)
Total	3.81 (0.43)	17.96 (5.01)	6.69 (2.13)

**Table 2:** Phenotypic correlations (top) and twin correlations for each zygosity (bottom) for Flow Proneness, Behavioral Inhibition, and Locus of Control.

Phenotypic correlations (95% confidence intervals)			
	Flow Proneness	Behavioral Inhibition	Locus of Control
BI	0.19 (0.16; 0.23)	-	-
LOC	0.18 (0.14; 0.22)	0.23 (0.21; 0.24)	-

	Twin correlations (95% confidence intervals)		
MZ	0.29 (0.12; 0.43)	0.45 (0.44; 0.50)	0.30 (0.24; 0.36)
DZ	0.09 (−0.05; 0.22)	0.14 (0.13; 0.18)	0.10 (0.06; 0.15)
MZ female	0.27 (0.05; 0.45)	0.51 (0.45; 0.57)	0.29 (0.20; 0.37)
MZ male	0.31 (0.04; 0.52)	0.37 (0.29; 0.44)	0.32 (0.22; 0.40)
DZ female	0.04 (−0.18; 0.26)	0.16 (0.08; 0.23)	0.11 (0.02; 0.20)
DZ male	−0.04 (−0.35; 0.28)	0.21 (0.12; 0.29)	0.06 (−0.04; 0.16)
DZOS	0.13 (−0.07; 0.32)	0.10 (0.04; 0.16)	0.12 (0.05; 0.19)

**Note.** MZ = Monozygotic; DZ = Dizygotic; DZOS = DZ opposite-sex.

### Genetic modeling

**Figure 1:**
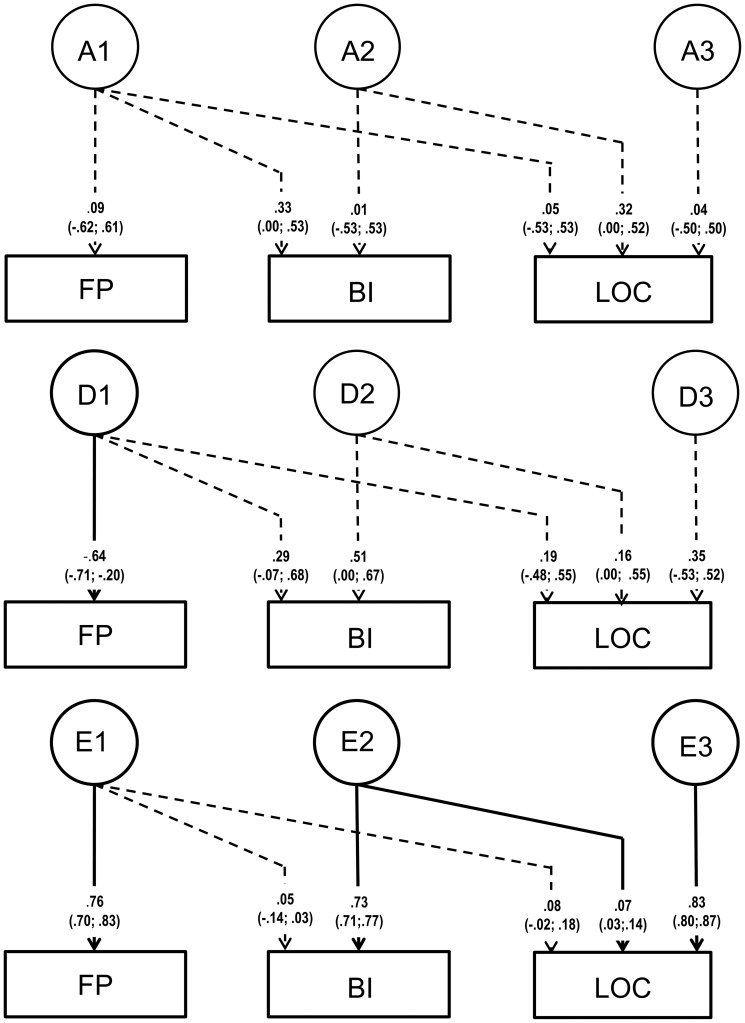
Trivariate ADE Cholesky decomposition for Flow Proneness (FP), Behavioural Inhibition (BI), and Locus of Control (LOC) showing non-significant pathways (dashed lines) for all additive (A) and most dominant (D) genetic influences indicating low power to distinguish between A and D.

Model fitting results of the trivariate analyses are shown in Table 3. The full ADE Cholesky decomposition indicated a lack of power to distinguish between A and D influences as the pathways were largely non-significant (Figure 1). The model fitting results (Table 3) of the reduced models further confirmed this, indicating that the DE model was more parsimonious than the AE and the ADE decompositions. However, as dominance without additive genetic influences is rather unlikely from a genetic perspective, a DE model is not plausible. For this reason we fitted a GE Cholesky decomposition [41]–[44] to obtain reliable estimates of the total genetic influences on the variance in and covariance between the three variables.

**Table 3:** Trivariate model fitting results for Flow Proneness, Behavioral Inhibition, and Locus of Control corrected for age and sex.

	AIC	−2LL	df	Δ −2LL	Δ - df	p-value
Cholesky decomposition – ADE	18193.32	64417.32	23112			
Cholesky decomposition – AE	18201.59	64437.59	23118	20.27	6	<0.01
Cholesky decomposition – DE	**18184.09**	**64420.09**	**23118**	**2.77**	**6**	**0.84**
Cholesky decomposition – GE	18184.23	64418.23	23117			

**Note.** GE and ADE decompositions (or sub-models) are not nested; therefore, their goodness of fit cannot be compared with each other. A = additive genetic; D = dominant genetic; E = non-shared environmental; G = genetic (A+D).

The goodness of fit of the AE, DE, and E Cholesky decompositions was compared to the full ADE decomposition.

**Figure 2:**
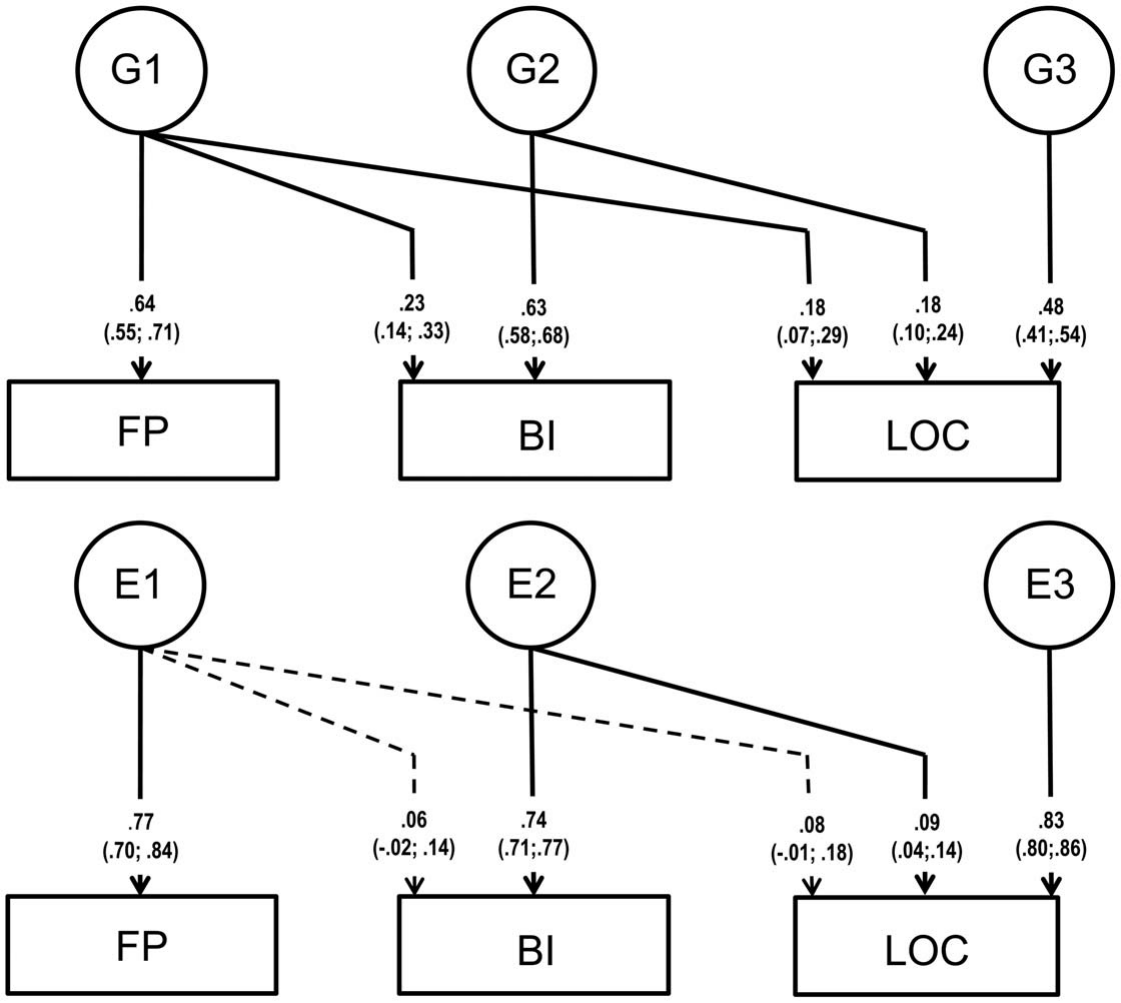
Full Cholesky decomposition showing genetic (G) and environmental (E) influences on the relationship between Flow Proneness (FP), Behavioral Inhibition (BI), and Locus of Control (LOC). Non-significant pathways in the model (*p*>0.05) were retained for completeness and are shown as dashed lines.

The trivariate GE Cholesky decompositions showed a good fit to the data (Figure 2), indicating that, although there were some shared genetic influences, most of the heritability was explained by specific genetic influences: One genetic factor shared between all three variables explained 12% and 11% of the total genetic variance of BI and LOC, respectively, with an additional genetic factor shared between BI and LOC explaining another 11% of the total genetic variance of LOC. Heritability estimates were moderate with .41, .45, and .30 for FP, BI and LOC, respectively. The remainder was mainly due to specific E influences indicating that almost all the covariance between the three variables was explained by shared genetic influences, i.e. only 1% of the total variance in LOC was explained by an E-factor shared with BI. The finding of large specific genetic factors was also reflected in the genetic correlations which were only moderate ranging between .33 (FP and LOC) and .42 (BI and LOC) with the environmental correlations being close to zero.

The univariate sex-limitation model for BI (Table S2) showed that while in males the entire heritability (.38) could be explained by additive genetic influences with a D-estimate of zero, in females A-influences were small (only explaining 13% of the total variance) and non-significant with significant dominant genetic influences explaining an additional 38% of the total variance. Although additive genetic effects could be equated, dominant genetic and non-shared environmental influences were significantly different, indicating that there is not only a significant difference in D-influences between sexes, but also in broad-sense heritability (G = A+D), with the heritability of BI in females being significantly higher (0.51) than in males (0.38).

## Discussion

The present study explored genetic and environmental influences on the relationships between FP, BI, and LOC in an adult twin sample. The phenotypic correlations between the three variables were low but significant and confirmed our hypothesis – individuals with an internal locus of control and a low behavioral inhibition were more likely to experience flow. Furthermore, the associations between FP, BI, and LOC appeared to be mainly genetically mediated. This suggests that the same set of genes predisposing individuals to feel in control of their destiny (internal LOC) and to be less inhibited (more extravert), also predisposes them to be more prone to have flow experiences. All three variables showed moderate heritabilities, with some indication of dominant genetic influences.

The presence of dominant genetic effects on all three studied traits was suggested both by the DZ correlations being less than half the MZ correlations, and by the trivariate modeling results, indicating a DE model as the most parsimonious. This is in line with evidence suggesting non-additive genetic influences on human personality traits [26], [47]–[49]. A high portion of genetic variation being non-additive might reflect past natural selection on a trait [50], [51]. Given the advantages of flow in terms of task performance, it is not difficult to imagine selection for higher predisposition to flow, especially in light of recent evidence suggesting that several personality traits have been under directional selection [52].

However, given that limited power resulted in non-significant estimates for most A and D influences in the full ADE Cholesky decompositions, we continued with a multivariate GE model in order to estimate total genetic effects with confidence intervals [41], [53]. The moderate heritabilities found for FP, LOC, and BI are in line with heritability estimates reported for the same, similar, and related personality traits [26]–[29], [31], [32], [34], [54]. In a previous study [30], we found slightly lower heritability estimates for domain-specific (work, maintenance, and leisure) flow proneness (h^2^ = .29–.35) compared to the heritability for overall flow proneness in the present study (h^2^ = .41). This may be due to the higher Cronbach's alpha for the full scale (.83) compared to the sub-scales (.61–.72). A lower Cronbach's alpha indicates a higher measurement error which would be reflected in inflated E-estimates, i.e. lower heritability.

Trivariate modeling showed that, in line with the rather low phenotypic correlations between the variables, the genetic (as well as environmental) influences on each trait were largely specific, with only little of the total genetic variance being explained by shared genetic influences. However, although shared genetic influences only explained little of the total genetic variance, they could explain almost the entire relationship (i.e. phenotypic correlation) between the three traits resulting in moderate genetic correlations.

Some of these common genes may be involved in dopaminergic functions. It is well known that dopaminergic neural systems play an important role in reward processing [55]–[58] and in impulse control [10], [59]–[62] which in turn has been shown to moderate the relationship between BI and anxiety [14], [63]. Also other cognitive functions related to locus of control have been shown to be modulated by dopamine pathways, such as executive functions and social cognition (for a review see [25]). Recent data from our group [64] show that flow proneness also is related to D2-dopamine receptor availability in the striatum (*r* = .41). Therefore, genetically based individual differences in dopaminergic function could explain some of the relationship between FP, BI and LOC.

Finally, univariate sex-limitation modeling for BI showed that there were not only differences in heritability of the trait between males and females (i.e. higher heritability in females), but also that unlike in males, in females dominant genetic influences played a significant role. In addition, given that male DZ twin correlations were more than half the MZ correlations, there also may be some common-environmental (C) influences on BI in males. To our knowledge, this is the first genetically informative study with sufficient power to specifically explore sex differences in BI. However, some indication for sex differences in the architecture of personality-related traits has been shown in past research [48], [65]–[71]. For example, Keller et al. [48] reported sex-specific genes for harm avoidance (fearful vs. carefree), a personality trait related to BI, although they did not find differences in overall heritability between sexes. It is important to note that we did not explore sex-specific influences as the DZ opposite-sex twin pair correlations were not significantly lower than the DZ same-sex correlations. However, DZ opposite-sex correlations were somewhat lower indicating that with additional power sex-specific influence may emerge.

As always, it is important to recognize the limitations of the present study. Our results cannot be generalized beyond the sample used – here consisting of middle-aged Swedish twins – so results may vary for different ethnicities and age groups. Correlations between the three variables were lower than expected, which, in combination with a relatively small sample size for the FP variable, resulted in limited power to distinguish between A and D effects in the full ADE Cholesky decompositions. This is further reflected in the relatively wide confidence intervals of the twin correlations and the variance component estimates. The limited sample size did also not allow for exploration of potential sex differences in the genetic architecture of the relationship of three variables. However, twin correlations for the five zygosity groups showed no indication for sex differences for FP and LOC, with the twin correlations being similar across the sexes in MZ and all three DZ groups. Furthermore, difficulties to derive precise estimates for genetic and environmental influences on the relationship between traits have been reported when based on samples of MZ and DZ twins only [72]. Finally, we did not explore gene-environment correlations and interactions and therefore cannot rule out those effects on our findings. Future studies with larger sample sizes and preferably extended twin design are needed to confirm our results and further explore potential sex differences in FP, BI, and LOC.

## Supporting Information

Table S1 Demographic information of individuals who only completed the paper-based questionnaire (not the online part) compared to those individuals who also filled out the online questionnaire.
(DOCX)

Table S2 Model fitting results for the univariate sex-limitation model of BI including same-sex twin pairs only.
(DOCX)
