# Supplementary material for: Genetic and Environmental Influences on the Relationship between Flow Proneness, Locus of Control and Behavioral Inhibition
Source: PLoS One. 2012 Nov 2;7(11):e47958. doi: 10.1371/journal.pone.0047958 (PMC3487896; doi:10.1371/journal.pone.0047958)
Supplement: Table S1 — Demographic information of individuals who only completed the paper-based questionnaire (not the online part) compared to those individuals who also filled out the online questionnaire. (DOCX) [file pone.0047958.s001.docx]

**Table S1.**  Demographic information of individuals who only completed the paper-based questionnaire (not the online part) compared to those individuals who also filled out the online questionnaire.

|  | Paper-based only (N = 8,257) | Online (N = 3,020) |
| --- | --- | --- |
| % male | 46% | 48% |
| Age - mean (SD) | 59.4 (4.5) | 58.4 (4.6) |
| BI - mean (SD) | 17.2 (4.9) | 18.0 (5.0) |
| LOC - mean (SD) | 6.4 (2.1) | 6.7 (2.1) |
