# Supplementary material for: Genetic and Environmental Influences on the Relationship between Flow Proneness, Locus of Control and Behavioral Inhibition
Source: PLoS One. 2012 Nov 2;7(11):e47958. doi: 10.1371/journal.pone.0047958 (PMC3487896; doi:10.1371/journal.pone.0047958)
Supplement: Table S2 — Model fitting results for the univariate sex-limitation model of BI including same-sex twin pairs only. (DOCX) [file pone.0047958.s002.docx]

**Table S2.** Model fitting results for the univariate sex-limitation model of BI including same-sex twin pairs only.

|  | A^2^ | D^2^ | E^2^ |
| --- | --- | --- | --- |
| Females | 0.13 (.00; .42) | **0.38 (.07; .56)** | **0.49 (.44; .54)** |
| Males | **0.38 (.08; .45)** | 0.00 (.00; .32) | **0.62 (.55; .69)** |

**Note.** Separate parameter estimates are shown for females and males with significant estimates highlighted in bold. A = additive genes; D = non-additive/dominant genetic; E = non-shared environment.
